# Contemporary use of SGLT2 inhibitors in heart failure patients with diabetes mellitus: a comparison of DPP4 inhibitors in a nationwide electric health database of the superaged society

**DOI:** 10.1186/s12933-022-01586-6

**Published:** 2022-08-13

**Authors:** Michikazu Nakai, Yoshitaka Iwanaga, Koshiro Kanaoka, Yoko Sumita, Yuichi Nishioka, Tomoya Myojin, Shinichiro Kubo, Katsuki Okada, Tsunenari Soeda, Tatsuya Noda, Yasushi Sakata, Tomoaki Imamura, Yoshihiko Saito, Satoshi Yasuda, Yoshihiro Miyamoto

**Affiliations:** 1 Department of Medical and Health Information Management, National Cerebral and Cardiovascular Center, 6-1 Kishibeshimmachi, Suita, Japan; 2 Department of Biostatistics, National Cerebral and Cardiovascular Center, Suita, Japan; 3 Department of Public Health, Health Management and Policy, Nara Medical University, Kashihara, Japan; 4 Department of Cardiovascular Medicine, Osaka University Graduate School of Medicine, Suita, Japan; 5 Department of Transformative System for Medical Information, Osaka University Graduate School of Medicine, Suita, Japan; 6 Department of Cardiovascular Medicine, Nara Medical University, Kashihara, Japan; 7 Department of Cardiovascular Medicine, Tohoku University Hospital, Tohoku University Graduate School of Medicine, Sendai, Japan; 8 Open Innovation Center, National Cerebral and Cardiovascular Center, Suita, Japan

**Keywords:** Diabetes mellitus, Nationwide Electric Health Database, One-year prognosis, An SGLT2 inhibitor, Superaged society

## Abstract

**Background:**

There is a lack of recent data reflecting the actual use of sodium-glucose cotransporter-2 (SGLT2) inhibitors for heart failure (HF) and type 2 diabetes (DM) in the superaged society. The present study investigated the association between the use of SGLT2 inhibitors and one-year prognosis in patients hospitalized across a broad spectrum of HF patients with DM in the superaged society using the Nationwide Electric Health Database in Japan.

**Methods:**

The patients hospitalized with the first episode of acute HF were identified from the National Database of Health Insurance Claims and Specific Health Checkups of Japan between April 2014 and March 2019. A cohort of 2,277 users of SGLT2 inhibitors and 41,410 users of the active comparator, dipeptidyl peptidase-4 (DPP4) inhibitors were compared. A propensity score-matched cohort study of 2,101 users of each inhibitor was also conducted. A multivariable multilevel mixed-effects survival model was conducted with adjustments, and hazard ratios (HRs) and 95% confidence intervals (CIs) were calculated.

**Results:**

Among 300,398 patients discharged with HF in 4,176 hospitals, 216,016 (71.9%) were 75 years or older, and 60,999 (20.3%) took antidiabetic medications. Among them, the patients treated with SGLT2 inhibitors were younger and had a more severe status than those treated with DPP4 inhibitors. Kaplan–Meier analysis showed that patients treated with SGLT2 inhibitors had a lower mortality risk and HF readmission. In propensity-matched cohorts, SGLT2 inhibitor use was associated with a lower risk of mortality and HF readmission than DPP-4 inhibitor use (HR [95% CI]; 0.70 [0.56, 0.89] and 0.52 [0.45, 0.61], respectively). Very elderly (≥ 75 years) patients showed similar results. Favorable effects were also observed across all age groups, including ≥ 75 years, in patients with coronary artery disease or atrial fibrillation and with concomitant β-blocker, diuretics, or insulin.

**Conclusion:**

The use of SGLT2 inhibitors at discharge was associated with a lower risk of one-year mortality and HF readmission in patients across a broad spectrum of HF with DM in the superaged society. The findings further support the benefits of using SGLT2 inhibitors in very elderly HF care and complement the current evidence.

**Supplementary Information:**

The online version contains supplementary material available at 10.1186/s12933-022-01586-6.

## Introduction

Diabetes mellitus (DM) is an important risk factor for developing heart failure (HF). Patients with DM have a 30% greater risk of requiring admission to the hospital due to HF than those without DM [1, 2]. Concurrent conditions, including coronary artery disease (CAD), hypertension, hyperlipidemia, and chronic kidney disease, may play a role in incident HF. Other mechanisms, such as metabolic dysfunction, inflammation, and adiposity, may also contribute to the risk of HF. Cardiovascular (CV) outcome trials with sodium–glucose cotransporter-2 inhibitor (SGLT2-I) showed a significant reduction in hospitalization for HF, as well as in CV and renal outcomes, compared with placebo in patients with DM, and they have been widely used in the clinical setting since 2014 [3, 4]. The meta-analysis shows that SGLT2-I is effective for both primary and secondary prevention of CV mortality or hospitalization for HF regardless of a history of HF [5]. However, clinicians still have concerns about the efficacy and safety of SGLT2-I in elderly patients with DM at risk of a variety of serious comorbidities. Although post-hoc analyses of randomized controlled trials have examined the CV benefits and safety of SGLT2-I in elderly adults (aged ≥ 65 years) with DM [6, 7], very elderly patients (≥ 75 years) have not been sufficiently represented in the trials. Some other studies have assured that the efficacy profile of SGLT2-I was unchanged by age based on the analysis of patients aged ≥ 65 years [8]; however, the efficacy and safety in very elderly DM patients are still unclear.

The average age of patients with HF in the UK and Japan is currently 77 and 78 years, respectively [9, 10]. In addition to the problems with the effectiveness and adverse effects of medications, elderly patients with HF have multiple comorbidities, typically 5 to 6 comorbidities in addition to HF [11]. The current evidence for medical therapy in HF is limited to elderly-centered HF patient groups. Therefore, the present study investigated the efficacy of SGLT2-I in patients across a broad spectrum of HF with DM to complement the current evidence and explore the best practice of SGLT2-I use in a superaged society. Notably, the impact of SGLT2-I use on 1-year clinical outcomes in the very elderly (≥ 75 years) and detailed associations with other concomitant medications were explored compared with the active comparator -dipeptidyl peptidase-4 inhibitor (DPP4-I)- using the current nationwide electronic health database in Japan.

## Methods

### Study population

A retrospective analysis was performed using the National Database of Health Insurance Claims and Specific Health Checkups of Japan (NDB). NDB is a Japanese administrative claims-based database created by the Ministry of Health, Labor, and Welfare of Japan. It includes claims on inpatient and outpatient services and prescriptions. Japan has a universal health coverage system, and the NDB covers approximately 98% of data on health care services provided by health care institutions, regardless of the type of insurance [12, 13]. The NDB includes two anonymized types of personal identification variables (ID1 and ID2) and the group of Nara Medical University has constructed a new patient-matching variable (ID0) with ID1 and ID2, which allows each patient to be followed up longitudinally using an individual claims data system in the NDB database [14]. The NDB includes sex, age groups [5-year groups], diagnostic codes based on the 10th revision of the International Statistical Classification of Diseases codes and outcome categories, codes for medical care received, drugs prescribed, and medical examinations performed, not including the results of the tests.

**Fig. 1:**
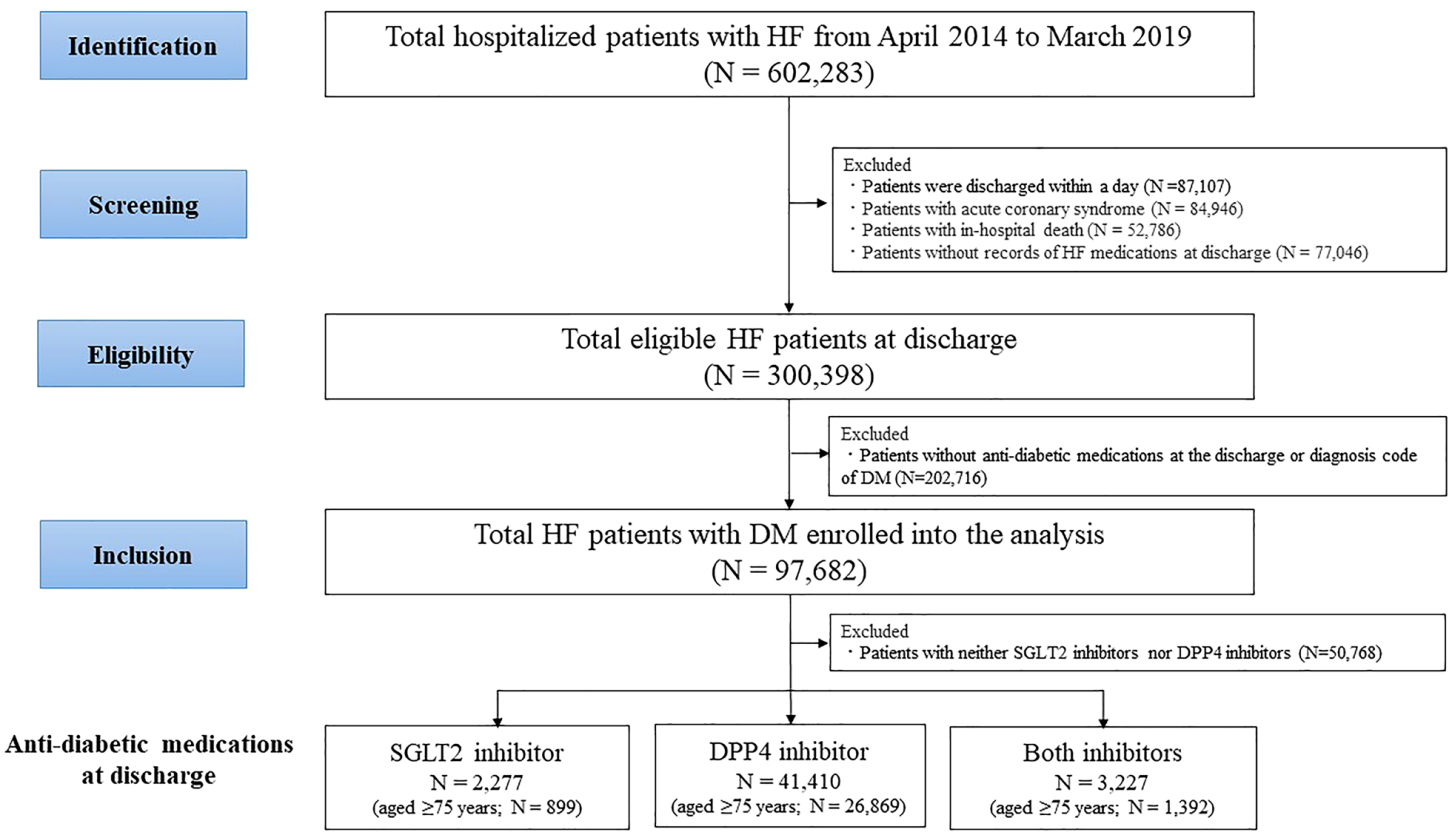
Study flow chart. DM, diabetes mellitus; DPP4, dipeptidyl peptidase-4; HF, heart failure: SGLT2, sodium-glucose cotransporter-2

Patients hospitalized with a first episode of acute HF from April 2014 to March 2019 were identified (Fig. 1). They were defined as patients with an emergency hospitalization and HF diagnostic code requiring intravenous diuretic (furosemide) or tolvaptan treatment within 1 day after admission. During hospitalization, they also underwent echocardiography and B-type natriuretic peptide (BNP) measurement. Patients who were discharged within 1 day, were diagnosed with acute coronary syndrome, or died in the hospital were excluded from the analysis. Patients who had no records of HF medications at discharge were also excluded. HF-related medications at discharge were defined as diuretics, β-blockers, angiotensin-converting enzyme inhibitors or angiotensin II receptor blockers (ACEIs/ARBs), mineralocorticoid receptor antagonists (MRAs), and digoxin. DM patients were defined as those with the diagnosis code as described in Additional file 1: Table S1 or antidiabetic medications at discharge, such as SGLT2-I, DPP4-I, sulfonylurea, metformin, insulin, glucagon-like peptide-1 agonist and others (α-glucosidase inhibitor, meglitinide, thiazolidine). Discharge prescriptions were defined as prescriptions made within 2 days before the discharge date with more than/equal to 7 days of prescription. Age was divided into 5 categories: < 54, 55–64, 65–74, 75–84, and > 85 years.

### Clinical outcomes

Clinical outcomes after discharge included all-cause mortality, HF readmission and all-cause readmission within 1 year, and were calculated from the discharge date. Death was investigated based on the logic-based method for determining mortality (sensitivity, 94.3%; specificity, 98.5%) [15]. HF readmission was defined as readmission with prescribing either intravenous diuretic (furosemide) or tolvaptan treatment within 1 day after admission and undergoing either echocardiography or BNP measurement during hospitalization in all-cause readmission. Our primary outcomes were in patients with SGLT2 inhibitor compared with DPP4 inhibitor in a cohort of propensity score matching. The secondary outcomes were in all patients with SGLT2 inhibitor (single or combined) compared with DPP4 inhibitor in a multivariable analysis. The third outcomes were with DM patients compared with Non-DM patients.

### Ethics statement

The study was conducted according to the 1964 Declaration of Helsinki and its later amendments. It was approved by the National Cerebral and Cardiovascular Center ethics committee (authorization number, R20110-2).

### Statistical analysis

Data are expressed as medians (interquartile range) for continuous variables. Categorical data are expressed as numbers (%). Univariate and multivariable multilevel mixed-effects survival models with institution as a random variable were constructed to observe the association between DM and non-DM as well as among SGLT2 inhibitor, DPP4 inhibitor and both inhibitor. Adjustment of multivariable model was sex and age category in Model 1. Model 2 was adjusted for sex, age category, inotropic agent use, procedures during hospitalization (respirator use, cardiac rehabilitation), CV medications at discharge (antiplatelet agents, anticoagulants, anti-arrhythmic agents, anti-hypertensive agents, and statins), HF medication at discharge (diuretics, β-blockers, ACEIs/ARBs, MRAs and digoxin) and HF comorbidity (valvular disease, cardiomyopathy, atrial fibrillation or atrial flutter [AF], CAD, and pulmonary hypertension, peripheral artery disease, chronic kidney disease, chronic obstructive pulmonary disease, and dementia) as described in Additional file 1: Table S1. Hazard ratios (HRs) and 95% confidence intervals (CIs) were calculated. Kaplan–Meier analysis was performed with the log-rank test. Propensity score-matching was conducted for the overall cohort of SGLT2 inhibitor and DPP4 inhibitor using the 1:1 nearest-neighbor matching method without replacement (caliper of 0.001) by logistic regression modeling, adjusted for the variables in Model 2. Standard mean differences were also calculated to evaluate the quality of the matching. Data were combined using structured query language, and statistical analysis was performed using Stata, version 16 (College Station, TX, USA).

## Results

### Comparison between DM and non-DM patients with HF

Among 300,398 HF patients enrolled, 71.91% were aged 75 years or older (Table 1). A total of 60,999 (20.31%) patients took antidiabetic medications at discharge. In patients with DM, those treated with β-blockers and ACEIs/ARBs were 60.31% and 63.63%, respectively. Regarding other CV medications, antiplatelets and anti-hypertensive agents were prescribed at discharge in 46.61% and 46.52% of the patients, respectively. Statins were prescribed in 44.07% of patients. They all were more frequently prescribed in DM patients than those without DM. The most common comorbidity among DM patients was CAD. DPP4-I was prescribed as an antidiabetic agent for most DM patients (73.18%). DM patients had a higher risk of mortality, and HF readmission than non-DM patients in multivariable analysis, adjusted for the variables in Model 2 (HR [95% CI]; 1.07 [1.03, 1.11] and 1.22 [1.19, 1.25], respectively, Additional file 1: Table S2). This finding was consistent in very elderly patients (≥ 75 years).

**Table 1:** Baseline clinical characteristics

	Total	Non-DM	DM	SGLT2-I	DPP4-I	Both
Number	300,398	202,716	97,682	2277	41,410	3227
Sex, male	150,597 (50.13%)	96,011 (47.36%)	54,586 (55.88%)	1563 (68.64%)	24,002 (57.96%)	2156 (66.81%)
Age category						
-54	17,714 (5.90%)	11,597 (5.72%)	6117 (6.26%)	450 (19.76%)	2248 (5.43%)	416 (12.89%)
55–64	19,976 (6.65%)	11,768 (5.81%)	8208 (8.40%)	359 (15.77%)	3531 (8.53%)	485 (15.03%)
65–74	46,692 (15.54%)	27,154 (13.40%)	19,538 (20.00%)	569 (24.99%)	8762 (21.16%)	934 (28.94%)
75–84	95,215 (31.70%)	61,353 (30.27%)	33,862 (34.67%)	643 (28.24%)	15,465 (37.35%)	999 (30.96%)
85-	120,801 (40.21%)	90,844 (44.81%)	29,957 (30.67%)	256 (11.24%)	11,404 (27.54%)	393 (12.18%)
Age at 75 years or older	216,016 (71.91%)	152,197 (75.08%)	63,819 (65.33%)	899 (39.48%)	26,869 (64.89%)	1392 (43.14%)
Medications at discharge						
Diuretics	256,829 (85.50%)	173,109 (85.39%)	83,720 (85.71%)	1835 (80.59%)	35,811 (86.48%)	2638 (81.75%)
β-blockers	163,884 (54.56%)	108,693 (53.62%)	55,191 (56.50%)	1666 (73.17%)	25,359 (61.24%)	2360 (73.13%)
ACEI/ARB	161,871 (53.89%)	105,231 (51.91%)	56,640 (57.98%)	1628 (71.50%)	26,431 (63.83%)	2339 (72.48%)
MRA	131,132 (43.65%)	90,625 (44.71%)	40,507 (41.47%)	1261 (55.38%)	17,111 (41.32%)	1692 (52.43%)
Digoxin	18,107 (6.03%)	12,871 (6.35%)	5236 (5.36%)	93 (4.08%)	2090 (5.05%)	140 (4.34%)
Triple therapy	50,075 (16.67%)	32,848 (16.20%)	17,227 (17.64%)	773 (33.95%)	8059 (19.46%)	1002 (31.05%)
Anti-platelets	87,256 (29.05%)	48,913 (24.13%)	38,343 (39.25%)	931 (40.89%)	19,474 (47.03%)	1583 (49.05%)
Anti-coagulants	125,101 (41.65%)	88,082 (43.45%)	37,019 (37.90%)	953 (41.85%)	16,360 (39.51%)	1346 (41.71%)
Anti-arrhythmic agents	21,722 (7.23%)	14,717 (7.26%)	7005 (7.17%)	218 (9.57%)	2963 (7.16%)	288 (8.92%)
Anti-hypertensive agents	109,097 (36.32%)	67,881 (33.49%)	41,216 (42.19%)	767 (33.68%)	19,581 (47.29%)	1253 (38.83%)
Anti-diabetic agents	60,999 (20.31%)					
SGLT2 inhibitors	5504 (1.83%)		5504 (5.63%)			
DPP4 inhibitors	44,637 (14.86%)		44,637 (45.70%)			
Sulfonylurea	10,307 (3.43%)		10,307 (10.55%)	141 (6.19%)	7219 (17.43%)	507 (15.71%)
Metformin	7159 (2.38%)	–	7159 (7.33%)	243 (10.67%)	4675 (11.29%)	638 (19.77%)
Insulin	17,678 (5.88%)	–	17,678 (18.10%)	486 (21.34%)	7738 (18.69%)	832 (25.78%)
GLP1 agonist	1014 (0.34%)	–	1014 (1.04%)	174 (7.64%)	36 (0.09%)	< 10
Others	13,820 (4.60%)		13,820 (14.15%)	201 (8.83%)	9306 (22.47%)	710 (22.00%)
Statin	74,588 (24.83%)	40,243 (19.85%)	34,345 (35.16%)	1072 (47.08%)	18,285 (44.16%)	1748 (54.17%)
Procedures during hospitalization						
Inotropic agents	51,647 (17.19%)	34,165 (16.85%)	17,482 (17.90%)	503 (22.09%)	7890 (19.05%)	760 (23.55%)
Ventilator use*	58,766 (19.56%)	36,649 (18.08%)	22,117 (22.64%)	660 (28.99%)	10,539 (25.45%)	1058 (32.79%)
Cardiac rehabilitation	126,986 (42.27%)	87,144 (42.99%)	39,842 (40.79%)	1311 (57.58%)	18,409 (44.46%)	1879 (58.23%)
Comorbidities						
Diabetes mellitus#	97,682 (32.52%)					
Valvular disease	48,604 (16.18%)	35,257 (17.39%)	13,347 (13.66%)	202 (8.87%)	4398 (10.62%)	298 (9.23%)
Cardiomyopathy	14,446 (4.81%)	10,316 (5.09%)	4130 (4.23%)	210 (9.22%)	1658 (4.00%)	194 (6.01%)
Atrial fibrillation/flutter	104,409 (34.76%)	75,910 (37.45%)	28,499 (29.18%)	706 (31.01%)	11,221 (27.10%)	937 (29.04%)
Coronary artery disease	83,207 (27.70%)	49,786 (24.56%)	33,421 (34.21%)	924 (40.58%)	14,281 (34.49%)	1396 (43.26%)
Pulmonary hypertension	4925 (1.64%)	3670 (1.81%)	1255 (1.28%)	31 (1.36%)	420 (1.01%)	28 (0.87%)
Peripheral artery disease	10,217 (3.40%)	4865 (2.40%)	5352 (5.48%)	64 (2.81%)	1887 (4.56%)	112 (3.47%)
Chronic kidney disease	47,250 (15.73%)	25,125 (12.39%)	22,125 (22.65%)	346 (15.20%)	10,009 (24.17%)	485 (15.03%)
COPD	11,753 (3.91%)	8434 (4.16%)	3319 (3.40%)	46 (2.02%)	1201 (2.90%)	79 (2.45%)
Dementia	21,244 (7.07%)	15,294 (7.54%)	5950 (6.09%)	43 (1.89%)	2159 (5.21%)	88 (2.73%)
Clinical Outcomes						
Hospitalization period, days	18.0 (12.0, 27.0)	17.0 (12.0, 27.0)	19.0 (13.0, 29.0)	17.0 (12.0, 25.0)	20.0 (14.0, 30.0)	18.0 (13.0, 27.0)
All-cause mortality	42,430 (14.12%)	29,506 (14.56%)	12,924 (13.23%)	131 (5.75%)	5229 (12.63%)	205 (6.35%)
≥ 75 years	38,066 (17.62%)	27,058 (17.78%)	11,008 (17.25%)	85 (9.45%)	4397 (16.36%)	138 (9.91%)
HF readmission	68,366 (22.76%)	44,049 (21.73%)	24,317 (24.89%)	296 (13.00%)	10,903 (26.33%)	457 (14.16%)
≥ 75 years	54,645 (25.30%)	37,177 (24.43%)	17,468 (27.37%)	155 (17.24%)	7715 (28.71%)	230 (16.52%)
All-cause readmission	161,309 (53.70%)	105,505 (52.05%)	55,804 (57.13%)	978 (42.95%)	24,566 (59.32%)	1547 (47.94%)
≥ 75 years	119,622 (55.38%)	82,339 (54.10%)	37,283 (58.42%)	426 (47.39%)	16,143 (60.08%)	690 (49.57%)

ACEI/ARB, angiotensin-converting enzyme inhibitor/angiotensin II receptor blocker; COPD, chronic obstructive pulmonary disease; DPP4-I, dipeptidyl peptidase-4 inhibitor; GLP1, glucagon-like peptide-1; HF, heart failure; MRA, mineralocorticoid receptor antagonist; SGLT2-I, sodium–glucose cotransporter-2 inhibitor

^*^Ventilator use includes noninvasive positive pressure ventilation. #Diabetes mellitus was defined as either having the diagnostic codes or anti-diabetic agents

### Comparison of SGLT2-I and DPP4-I

Comparing the DM patients who had SGLT2-I and DPP4-I in the crude cohort (Table 1), patients with SGLT2-I showed a higher percentage of males and were younger. They had more HF medications such as β-blockers (SGLT2-I: 73.17% and DPP4-I: 61.24%), ACEIs/ARBs (SGLT2-I: 71.50% and DPP4-I: 63.83%), and MRAs (SGLT2-I: 55.38% and DPP4-I: 41.32%). During hospitalization, 22.09% and 28.99% of SGLT2-I patients had an inotropic agent and ventilator use, respectively, and 57.58% underwent cardiac rehabilitation. Patients treated with both inhibitors showed similar characteristics to those treated with SGLT2-I. Baseline characteristics after propensity matching are shown in Additional file 1: Table S3.

**Fig. 2:**
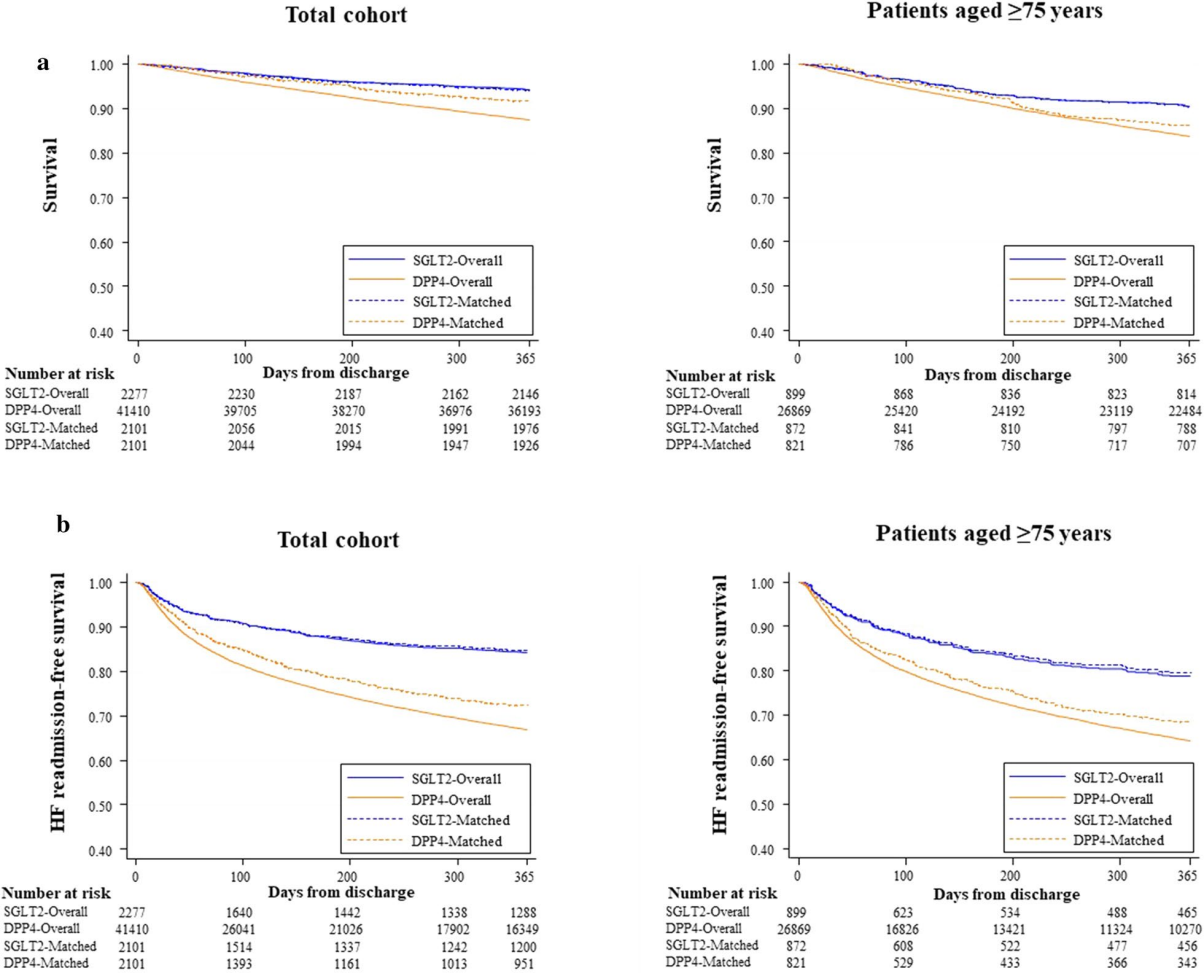
Kaplan–Meier analysis of all-cause mortality (**a**) and HF readmission (**b**) for SGLT2 and DPP4 inhibitor use at discharge in overall patients and in patients aged ≥ 75 years. DPP4-I, dipeptidyl peptidase-4 inhibitor; HF, heart failure: SGLT2-I, sodium-glucose cotransporter-2 inhibitor

Compared with DPP4-I, Kaplan–Meier analysis showed that the patients with SGLT2-I were associated with lower mortality and HF readmission rates in both the original and matched datasets. Very elderly patients (≥ 75 years) showed similar results (Fig. 2). With patients with DPP4-I as the reference group, multivariable logistic analysis after adjustment (Model 2) showed that the HR (95% CI) for mortality with SGLT2-I patients was 0.65 (0.55, 0.78) in the original dataset and 0.65 (0.49, 0.91) in the matched dataset (Table 2). The HR (95% CI) for HF readmission with SGLT2-I patients was 0.49 (0.43, 0.55) in the original dataset and 0.46 (0.36, 0.59) in the matched dataset. Similarly, in a sub-analysis of very elderly patients, the HR (95% CI) for mortality with SGLT2-I patients was 0.63 (0.51,0.79) in the original dataset and 0.63 (0.43, 0.92) in the matched dataset. The HR (95% CI) for HF readmission with SGLT2-I patients was 0.54 (0.46, 0.63) in the original dataset and 0.46 (0.33, 0.64) in the matched dataset. In addition, the use of both inhibitors was associated with better outcomes compared with DPP4-I only. The results of all-cause readmission were similar (Additional file 1: Figure S1 and Table 2).

**Table 2:** Univariate/multivariable multilevel logistic analysis for mortality and readmission in the prevalent DM cohort

	Univariate	Model 1	Model 2	Matched model
	HR (95% CI)	HR (95% CI)	HR (95% CI)	HR (95% CI)
Mortality				
Overall				
DPP4 inhibitor	Ref	Ref	Ref	Ref
SGLT2 inhibitor	0.44 (0.37,0.52)	0.62(0.52,0.74)	0.67 (0.56,0.80)	0.70 (0.56,0.89)
Both inhibitors	0.49 (0.42,0.56)	0.65 (0.56,0.74)	0.71 (0.62,0.82)	
Patients ≥ 75 years				
DPP4 inhibitor	Ref	Ref	Ref	Ref
SGLT2 inhibitor	0.56 (0.45,0.69)	0.59 (0.48,0.74)	0.64 (0.52,0.80)	0.68 (0.51,0.90)
Both inhibitors	0.58 (0.49,0.69)	0.63 (0.53,0.75)	0.69 (0.58,0.82)	
HF readmission				
Overall				
DPP4 inhibitor	Ref	Ref	Ref	Ref
SGLT2 inhibitor	0.43 (0.38,0.48)	0.48 (0.42,0.54)	0.50 (0.44,0.56)	0.52 (0.45,0.61)
Both inhibitors	0.49 (0.45,0.54)	0.53 (0.49,0.59)	0.56 (0.51,0.62)	
Patients ≥ 75 years				
DPP4 inhibitor	Ref	Ref	Ref	Ref
SGLT2 inhibitor	0.54 (0.46,0.63)	0.53 (0.45,0.62)	0.54 (0.46,0.64)	0.59 (0.47,0.74)
Both inhibitors	0.53 (0.46,0.60)	0.52 (0.46,0.60)	0.55 (0.48,0.62)	
All-cause readmission				
Overall				
DPP4 inhibitor	Ref	Ref	Ref	Ref
SGLT2 inhibitor	0.63 (0.59,0.67)	0.65 (0.61,0.69)	0.68 (0.63,0.72)	0.72 (0.66,0.79)
Both inhibitors	0.74 (0.70,0.78)	0.76 (0.72,0.80)	0.78 (0.74,0.83)	
Patients ≥ 75 years				
DPP4 inhibitor	Ref	Ref	Ref	Ref
SGLT2 inhibitor	0.70 (0.64,0.77)	0.69 (0.63,0.76)	0.71 (0.64,0.78)	0.76 (0.65,0.87)
Both inhibitors	0.76 (0.71,0.82)	0.75 (0.69,0.81)	0.77 (0.71,0.83)	

In Model 1, HR was adjusted for age category and sex. In Model 2, HR was adjusted for age category, sex, and other 22 factors

*CI*, confidence interval; *DPP4,* dipeptidyl peptidase-4; *HF,* heart failure; *HR,* hazard ratio; *SGLT2,* sodium–glucose cotransporter-2

### Stratified analysis in the matched cohort

**Fig. 3:**
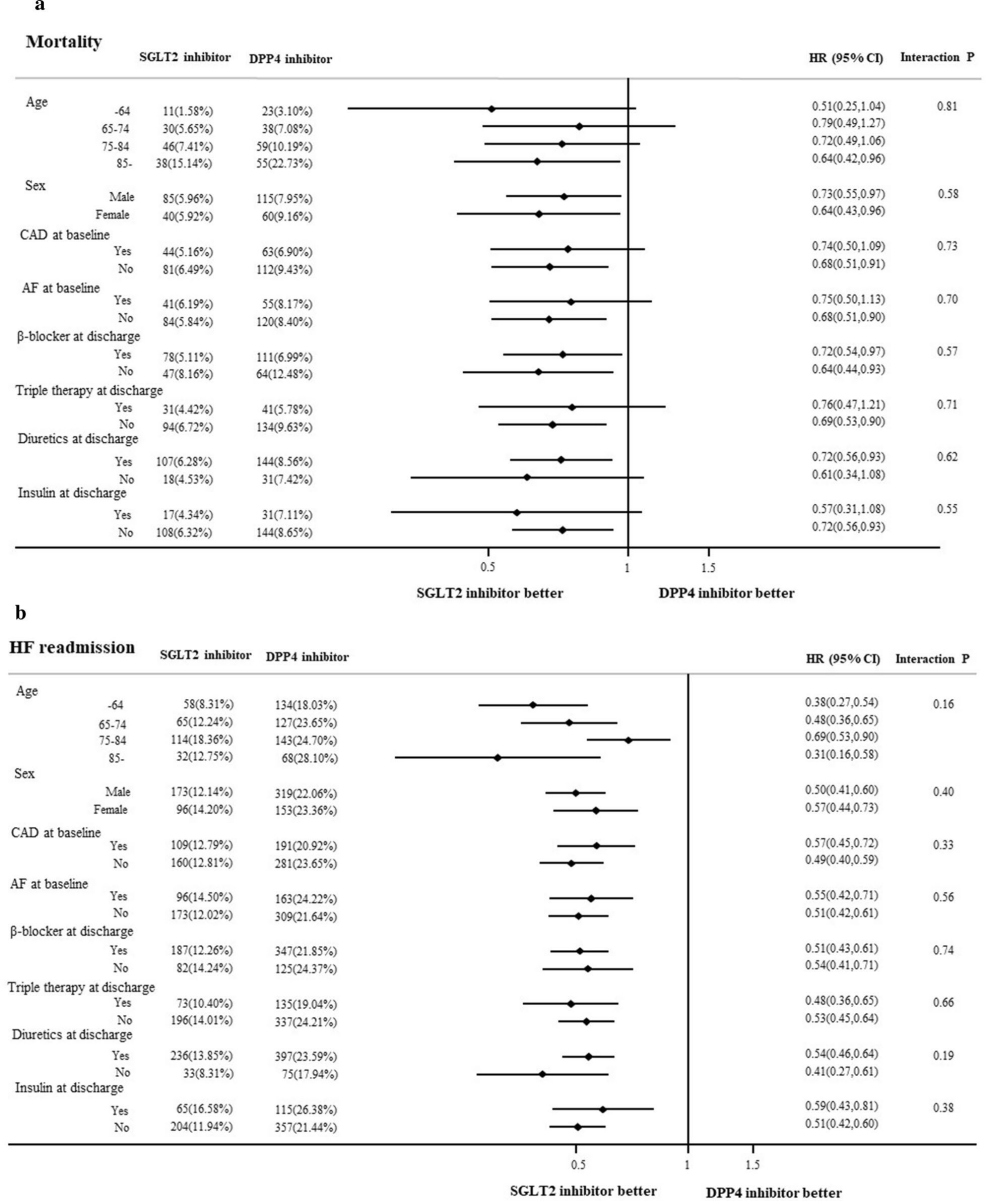
Stratified analysis for all-cause mortality (**a**) and HF readmission (**b**) in the propensity-matched cohort. AF, atrial fibrillation or atrial flutter; CAD, coronary artery disease; CI, confidence interval; DPP4, dipeptidyl peptidase-4; HF, heart failure; SGLT2, sodium-glucose cotransporter-2

In stratified analyses based on a propensity score–matching dataset, there was no interaction among most baseline characteristics, such as age classification, sex, comorbidities of CAD and AF, and concomitant drugs, such as β-blockers, diuretics, and insulin, by SGLT2-I and DPP4-I use (Fig. 3). Very elderly patients including those aged ≥ 75 years, patients with CAD or AF, and patients with concomitant β-blocker, diuretics, and insulin prescriptions, showed a decreased risk of mortality and HF readmission a similar manner. The results of all-cause readmission were also consistent (Additional file 1: Figure S2).

## Discussion

In the present study, we analyzed a nationwide real-world dataset of 300,398 patients hospitalized for acute HF, where 72% of patients were aged ≥ 75 years. Among 60,999 (20.3%) patients taking antidiabetic medications, 2,277 patients with SGLT2-I were younger and had a more severe HF status than 41,410 patients with DPP4-I. In both the original and propensity-score matching datasets, SGLT2-I use was associated with a lower risk of mortality, HF readmission, and all-cause readmission in a year than DPP-4 I use. Similar favorable effects were observed in patients across all age classes, with CAD or AF, and with concomitant use of β-blockers, diuretics, or insulin.

### Clinical benefits of SGLT2-I in DM and HF

Multiple randomized controlled clinical trials have shown a positive impact of SGLT2-I on the risk reduction of CV mortality and HF hospitalization in patients with DM. Real-world observational studies in DM patients have also supported the effectiveness [16, 17]. Based on these studies, SGLT2-I represents a new therapeutic drug for DM patients at risk for HF and HF. The benefits of SGLT2-I have recently been demonstrated in patients with established HF with reduced ejection fraction (HFrEF) irrespective of DM status [18]. In addition, the EMPEROR-Preserved study showed significantly reduced CV mortality or hospitalization for HF in patients taking empagliflozin compared to those taking placebo, suggesting a promising future for SGLT2-I in HF with preserved ejection fraction (HFpEF) [19]. The present results from routine clinical practice complement the evidence from the SGLT2-I outcome trials, performed in a highly selected population. It is important to be a very elderly-centered HF cohort since elderly patients, and patients with recent HF hospitalization are underrepresented in clinical trials in DM and HF. The present study reassures clinicians that, in real-world data, the treatment effect of SGLT2-I was similar across all age groups, including patients > 75 years of age [20].

It is critical, especially in managing of elderly patients, for physicians to identify patients with whom this drug is appropriate. The stratified analyses in our study showed beneficial effects irrespective of comorbidities such as AF and CAD. Additionally, the concomitant drugs for HF and DM did not affect the beneficial effects of SGLT2-I. The combination with diuretics or insulin did not have a detrimental effect, and the combinations of SGLT2-I and β-blockers may cause a lower risk of mortality. The favorable effects of SGLT2-I were consistent across both comorbidities and concomitant drugs may suggest a wide range of use and benefits in patients with a broad spectrum of DM and HF.

### Comparison with DPP4-I

DPP4-I has been demonstrated to be a safe pharmacological class with a neutral effect on CV outcomes, including HF hospitalization [21]. The use represents a safe and effective therapeutic choice in elderly patients with DM, HF, and other comorbidities [22], and DPP4-I was chosen as an active comparator in the present study because it has been widely used in Japan. Our results showed that SGLT2-I was superior in terms of a-year mortality and HF readmission. In a large Scandinavian cohort of DM patients, SGLT2-I use compared with DPP4-I use was associated with a reduced risk of HF and all-cause mortality [23]. Moreover, the clinical data of DM patients across 13 countries, including Japan, have shown that lower mortality and HF hospitalization were associated with SGLT2-I use (HR [95% CI]: 0.59 [0.52–0.67] and 0.69 [0.61–0.77], respectively) [24]. Furthermore, the same nationwide population-based study has shown the effectiveness of SGLT2 inhibitors in older general patients with DM [25]. Although there are many comparative studies on such DM patients, few studies have compared the effectiveness of SGLT2-I with DPP4-I in real-world clinical data of HF patients. This study showed the efficacy of SGLT2-I as an antidiabetic drug from patients’ viewpoints across a broad spectrum of HF. There are substantial differences in clinical outcomes between general DM population and DM population with HF hospitalization. The effectiveness of SGLT2 inhibitors may be stronger in HF population to whom more clinical events occur, and the SGLT2 inhibitors were effective even in very-elderly patients. It complemented the practical use of HF patients with DM in routine clinical practice. However, there is a report that the frequency of ketoacidosis was increased compared with that of DPP4-I use in elderly DM patients [26]. Caution and further research on this issue are necessary.

### Limitations

Our study has several limitations. First, the present study was performed in a cohort with a broad spectrum of HF, i.e., in patients with nonspecific EF. Since NDB is an administrative dataset and clinical data such as ejection fraction were unavailable, it was impossible to differentiate between HFrEF and HFpEF in the present analysis. However, a recent study has shown that the effects of empagliflozin have been evaluated across the full spectrum of EF (< 65%) in patients with HF [27]. Second, the safety of SGLT2-I was not investigated in the present study because NDB is a limited data source for evaluating of drug side effects. However, the finding that SGLT2-I was associated with a reduced risk of all-cause readmission may suggest the overall safety profile of this drug in the present population. Elderly patients are more likely to have adverse events and drug intolerance, and further study is necessary to investigate the detailed safety profile of this drug in very elderly patients. Last, regarding the effect of medications on clinical outcomes, it might be challenging to fully adjust for disease severity, the difference in individual in-hospital treatment, and the indication for medication by the provider, even with sophisticated statistical techniques. NDB did not provide information about DM severity (hemoglobin A1c), chronic kidney disease stage (glomerular filtration rate and proteinuria), or evaluations of the quality of life and frailty, which may significantly impact on the patient’s prognosis, especially in very elderly patients. The conclusions regarding the efficacy of SGLT2-I treatment should be interpreted with caution at this point.

## Conclusion

The use of SGLT2 inhibitors at discharge was associated with a lower risk of one-year mortality and HF readmission in patients across a broad spectrum of HF with DM using the current nationwide electronic health database in Japan. The findings further support the clinical benefits of SGLT2-I in the very elderly (≥ 75 years) HF care and the best practice of SGLT2-I use in a superaged society.

## Supplementary Information

**Additional file 1: Figure S1.** Kaplan–Meier analysis of all-cause readmission for SGLT2 and DPP4 inhibitor use at discharge in overall patients and in patients aged ≥75 years. **Figure S2.** Stratified analysis for all-cause readmission in the propensity-matched cohort. **Table S1.** Definition and codes used in baseline characteristics. **Table S2**. Univariate/multivariable multilevel logistic analysis for mortality and readmissions. **Table S3. **Baseline characteristics after propensity matching.

## Data Availability

Data may be obtained from a third party and are not publicly available.

## Abbreviations

ACEI/ARB: Angiotensin-converting enzyme inhibitor or angiotensin II receptor blocker
AF: Atrial fibrillation or atrial flutter
BNP: B-type natriuretic peptide
CAD: Coronary artery disease
CI: Confidence interval
CV: Cardiovascular
DM: Diabetes mellitus
DPP4-I: Dipeptidyl peptidase-4 inhibitor
HF: Heart failure
HFpEF: Heart failure with preserved ejection fraction
HFrEF: Heart failure with reduced ejection fraction
HR: Hazard ratio
MRA: Mineralocorticoid receptor antagonist
NDB: National Database of Health Insurance Claims and Specific Health Checkups of Japan
SGLT2-I: Sodium–glucose cotransporter-2 inhibitor
