# Supplementary material for: Contemporary use of SGLT2 inhibitors in heart failure patients with diabetes mellitus: a comparison of DPP4 inhibitors in a nationwide electric health database of the superaged society
Source: Cardiovasc Diabetol. 2022 Aug 13;21:157. doi: 10.1186/s12933-022-01586-6 (PMC9375946; doi:10.1186/s12933-022-01586-6)
Supplement: Supplementary file 1 — Additional file 1: Figure S1. Kaplan–Meier analysis of all-cause readmission for SGLT2 and DPP4 inhibitor use at discharge in overall patients and in patients aged ≥75 years. Figure S2. Stratified analysis for all-cause readmission in the propensity-matched cohort. Table S1. Definition and codes used in baseline characteristics. Table S2. Univariate/multivariable multilevel logistic analysis for mortality and readmissions. Table S3. Baseline characteristics after propensity matching. [file 12933_2022_1586_MOESM1_ESM.docx]

**
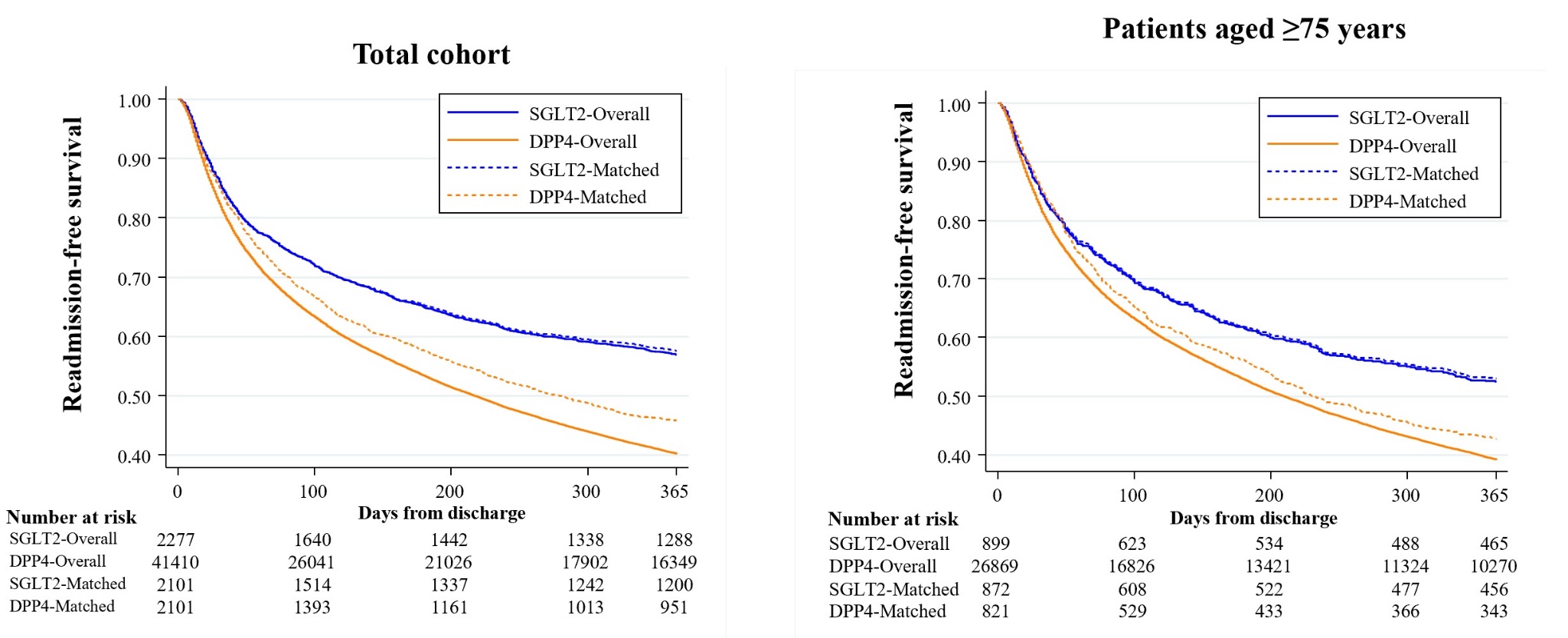
Figure S1. Kaplan–Meier analysis of all-cause readmission for SGLT2 and DPP4 inhibitor use at discharge in overall patients and in patients aged ≥75 years**

DPP4-I, dipeptidyl peptidase-4 inhibitor; SGLT2-I, sodium-glucose cotransporter-2 inhibitor

**Figure S2. Stratified analysis for all-cause readmission in the propensity-matched cohort**

**
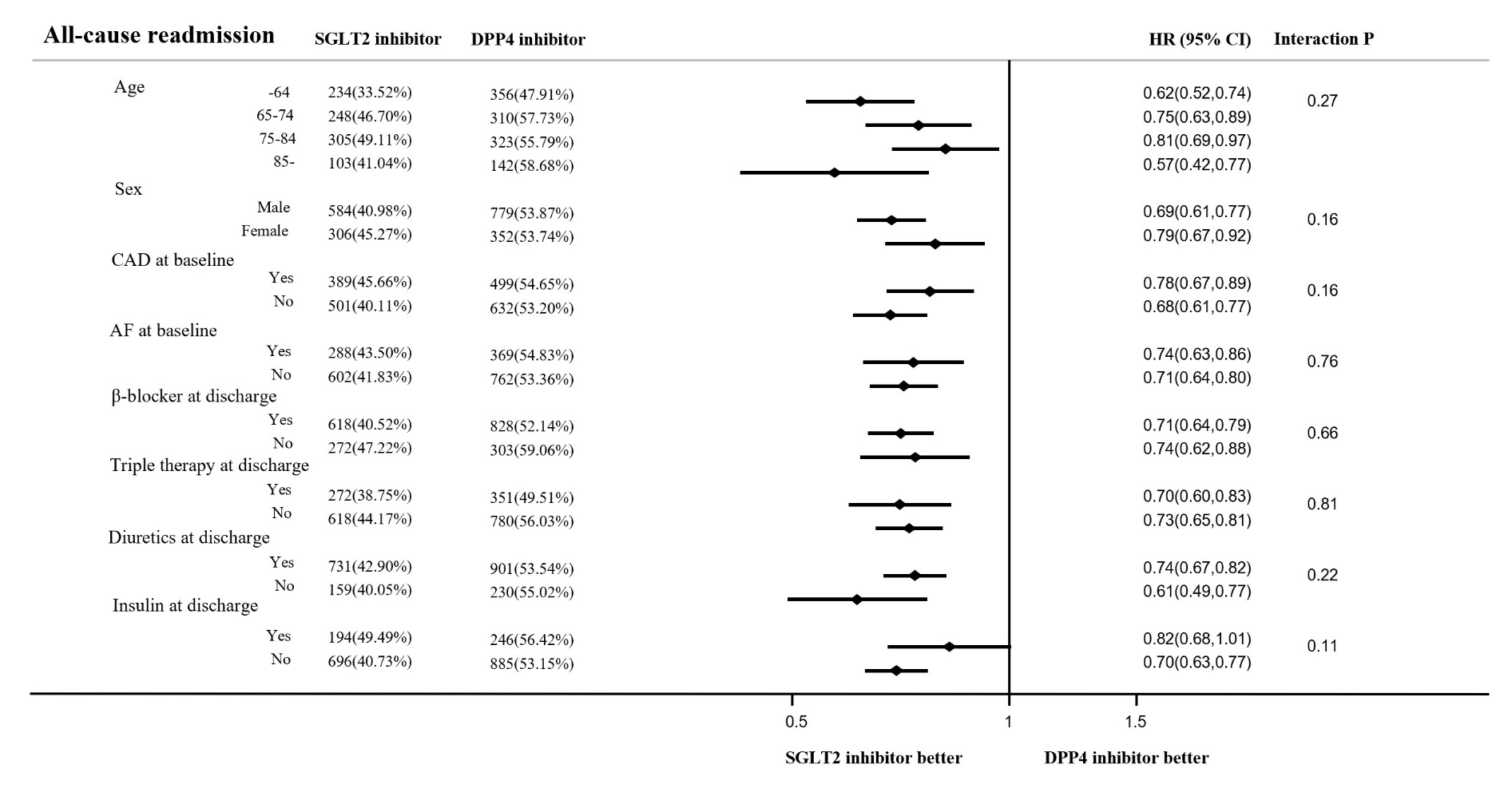
**

AF, atrial fibrillation or atrial flutter; CAD, coronary artery disease; CI, confidence interval; DPP4, dipeptidyl peptidase-4; HF, heart failure; SGLT2, sodium-glucose cotransporter-2

**Table S1. Definition and codes used in baseline characteristics**

| Key condition | Definitions | ICD10 code |
| --- | --- | --- |
|  |  |  |
| **Discharged medicine** |  |  |
| diuretics | Loop diuretics, tolvaptan |  |
| Anti-platelets | Aspirin, clopidogrel, cilostazol, ticlopidine, prasugrel |  |
| Anti-coagulants | Warfarin, dabigatran, rivaroxaban, apixaban, edoxaban |  |
| Anti-arrhythmic agents | Quinidine, disopyramide, cibenzoline, pilsicainide, pilmenol, flecainide, procainamide, propafenone, mexiletine, lidocaine, amiodarone, sotalol, nifekalant, bepridil |  |
| Anti-hypertensive agents | Calcium channel blocker, thiazide |  |
| Anti-diabetic agents | Dipeptidyl peptidase-4 inhibitor, sodium–glucose cotransporter 2 inhibitor, sulfonylurea, alpha-glucosidase, glipizide, thiazolidine, metformin, insulin, glucagon-like peptide-1 receptor agonist |  |
|  |  |  |
| **Comorbidities** |  |  |
| Valvular disease | Defined from diagnosis codes | I05-I08, I34-I37 |
| Cardiomyopathy | Defined from diagnosis codes | I42 |
| Atrial fibrillation/flutter | Defined from diagnosis codes | I48 |
| Coronary artery disease | Defined from diagnosis codes | I20, I25 (excluding I25.4) |
| Pulmonary hypertension | Defined from diagnosis codes | I27 |
| Diabetes mellitus | Defined from diagnosis codes or anti-diabetic medications | E10-E14 |
| COPD | Defined from diagnosis codes | J43, J44 |
| Dementia | Defined from diagnosis codes | G10, G30, G238, G310, G318, F01, F03, F050, F051, F107, B220, E756 |
| Peripheral artery disease | Defined from diagnosis codes | I70, I743, I744, I745, I749 |
| Chronic kidney disease | Defined from diagnosis codes | N17-N19, N391, R80, E112 |

COPD, chronic obstructive pulmonary disease; ICD10, International Classification of Diseases,10^th^ revision

**Table S2**. **Univariate/multivariable multilevel logistic analysis for mortality and readmissions**

|  | **Univariate** | **Model 1** | **Model 2** |
| --- | --- | --- | --- |
|  | **HR (95% CI)** | **HR (95% CI)** | **HR (95% CI)** |
| **Mortality** |  |  |  |
| Total patients |  |  |  |
| DM/non-DM | 0.89 (0.87,0.91) | 1.03 (1.01,1.06) | 1.03 (1.01,1.06) |
| Patients ≥75 years |  |  |  |
| DM/non-DM | 0.96 (0.94,0.98) | 1.02 (1.00,1.05) | 1.03 (1.00,1.06) |
| Patients ≥85 years |  |  |  |
| DM/non-DM | 1.01 (0.98,1.04) | 1.00 (0.97,1.03) | 1.01 (0.98,1.04) |
|  |  |  |  |
| **HF readmission** |  |  |  |
| Total patients |  |  |  |
| DM/non-DM | 1.20 (1.19,1.22) | 1.27 (1.25,1.29) | 1.14 (1.12,1.16) |
| Patients ≥75 years |  |  |  |
| DM/non-DM | 1.17 (1.15,1.19) | 1.17 (1.15,1.19) | 1.10 (1.07,1.12) |
| Patients ≥85 years |  |  |  |
| DM/non-DM | 1.10 (1.07,1.13) | 1.09 (1.06,1.12) | 1.04 (1.01,1.07) |
|  |  |  |  |
| **All-cause readmission** |  |  |  |
| Total patients |  |  |  |
| DM/non-DM | 1.15 (1.14,1.16) | 1.17 (1.16,1.18) | 1.07 (1.06,1.09) |
| Patients ≥75 years |  |  |  |
| DM/non-DM | 1.13 (1.11,1.14) | 1.11 (1.10,1.13) | 1.05 (1.04,1.07) |
| Patients ≥85 years |  |  |  |
| DM/non-DM | 1.08 (1.06,1.10) | 1.07 (1.05,1.09) | 1.03 (1.01,1.05) |

CI, confidence interval; DM, diabetes mellitus; HF, heart failure; HR, hazard ratio

In Model 1, HR was adjusted for age category and sex. In Model 2, HR was adjusted for age category, sex, and other 22 factors.

**Table S3. Baseline characteristics after propensity matching**

|  | **SGLT2-I** | **DPP4-I** | **Standardized difference** |
| --- | --- | --- | --- |
| **N** | 2101 | 2101 |  |
| **Sex, male** | 1425 (67.82%) | 1446 (68.82%) | 0.021 |
| **Age category** |  |  | 0.039 |
| **-54** | 369 (17.56%) | 370 (17.61%) |  |
| **55-64** | 329 (15.66%) | 373 (17.75%) |  |
| **65-74** | 531 (25.27%) | 537 (25.56%) |  |
| **75-84** | 621 (29.56%) | 579 (27.56%) |  |
| **85-** | 251 (11.95%) | 242 (11.52%) |  |
| **Age at 75 years or older** | 872 (41.50%) | 821 (39.08%) | 0.049 |
|  |  |  |  |
| **Medications at discharge** | | | |
| **Diuretics** | 1704 (81.10%) | 1683 (80.10%) | 0.025 |
| **β-Blockers** | 1525 (72.58%) | 1588 (75.58%) | 0.068 |
| **ACEI/ARB** | 1496 (71.20%) | 1496 (71.20%) | 0.000 |
| **MRA** | 1161 (55.26%) | 1192 (56.73%) | 0.029 |
| **Digoxin** | 84 (4.00%) | 95 (4.52%) | 0.025 |
| **Triple therapy** | 702 (33.41%) | 709 (33.75%) | 0.007 |
| **Anti-platelets** | 862 (41.03%) | 875 (41.65%) | 0.012 |
| **Anti-coagulants** | 885 (42.12%) | 898 (42.74%) | 0.012 |
| **Anti-arrhythmic agents** | 194 (9.23%) | 234 (11.14%) | 0.062 |
| **Anti-hypertensive agents** | 713 (33.94%) | 700 (33.32%) | 0.013 |
| **Anti-diabetic agents** |  |  |  |
| **Sulfonylurea** | 126 (6.00%) | 141 (6.71%) | 0.029 |
| **Metformin** | 219 (10.42%) | 228 (10.85%) | 0.013 |
| **Insulin** | 392 (18.66%) | 436 (20.75%) | 0.052 |
| **GLP1 agonist** | 24 (1.14%) | 20 (0.95%) | 0.018 |
| **Others** | 174 (8.28%) | 191 (9.09%) | 0.028 |
| **Statin** | 973 (46.31%) | 991 (47.17%) | 0.017 |
|  |  |  |  |
| **Procedures during hospitalization** | | | |
| **Inotropic agents** | 455 (21.66%) | 494 (23.51%) | 0.044 |
| **Ventilator use*** | 606 (28.84%) | 666 (31.70%) | 0.062 |
| **Cardiac rehabilitation** | 1195 (56.88%) | 1241 (59.07%) | 0.044 |
|  |  |  |  |
| **Comorbidities** | | | |
| **Valvular disease** | 191 (9.09%) | 205 (9.76%) | 0.022 |
| **Cardiomyopathy** | 182 (8.66%) | 209 (9.95%) | 0.044 |
| **Atrial fibrillation/flutter** | 662 (31.51%) | 673 (32.03%) | 0.011 |
| **Coronary artery disease** | 852 (40.55%) | 913 (43.46%) | 0.058 |
| **Pulmonary hypertension** | 28 (1.33%) | 33 (1.57%) | 0.019 |
| Peripheral artery disease | 60 (2.86%) | 62 (2.95%) | 0.005 |
| Chronic kidney disease | 306 (14.56%) | 310 (14.75%) | 0.005 |
| COPD | 46 (2.19%) | 54 (2.57%) | 0.024 |
| Dementia | 41 (1.95%) | 55 (2.62%) | 0.044 |
|  |  |  |  |
| **Clinical Outcomes** | | | |
| **Hospitalization period** | 17.0 (12.0, 25.0) | 19.0 (13.0, 28.0) | 0.119 |
| **All-cause mortality** | 125 (5.95%) | 175 (8.33%) | 0.092 |
| **HF readmission** | 269 (12.80%) | 472 (22.47%) | 0.255 |
| **All-cause readmission** | 890 (42.36%) | 1131 (53.83%) | 0.231 |

ACEI/ARB, angiotensin-converting enzyme inhibitor/angiotensin II receptor blocker; DM, diabetes mellitus; DPP4-I, dipeptidyl peptidase-4 inhibitor; GLP1, glucagon-like peptide-1; HF, heart failure; MRA, mineralocorticoid receptor antagonist; SGLT2-I, sodium–glucose cotransporter-2 inhibitor; COPD, chronic obstructive pulmonary disease

*Ventilator use includes noninvasive positive pressure ventilation.
